# *Notes from the Field:* Occupational Carbon Monoxide Exposure in an Industrial Kitchen Facility ― Wisconsin, 2017

**DOI:** 10.15585/mmwr.mm6728a5

**Published:** 2018-07-20

**Authors:** Erica Wilson, Carrie Tomasallo, Jonathan Meiman

**Affiliations:** ^1^Epidemic Intelligence Service, CDC; ^2^Bureau of Environmental and Occupational Health, Wisconsin Department of Health Services, Madison, Wisconsin.

On September 6, 2017, the Wisconsin Poison Center was contacted by emergency department (ED) health care providers at two hospitals who requested consultation for management of multiple patients with occupational carbon monoxide (CO) exposure. CO is an odorless, colorless gas that kills approximately 400 persons annually in the United States (*1*). The Wisconsin Division of Public Health received a surveillance alert from the Wisconsin Poison Center and launched an investigation to characterize the exposures and provide public health recommendations. The Wisconsin Division of Public Health conducted key informant interviews with emergency responders and reviewed ED medical records.

According to key informant interviews, first responders had received a call on September 5 from a manufacturer of frozen appetizers who suspected a CO leak in the manufacturing facility. CO levels were obtained in multiple areas of the facility and reached a peak of 313 ppm (National Institute for Occupational Safety and Health ceiling recommended exposure limit is 200 ppm) in an area of the facility with gas-burning fryers. The facility was evacuated, and natural gas was turned off. Forty-five employees were triaged on site; 37 were transported to local EDs for assessment and treatment for CO exposure. Four symptomatic employees who had gone home sick were instructed to proceed to the nearest ED for evaluation.

During September 6–October 3, the Wisconsin Division of Public Health obtained medical records for 40 persons, including 36 (97%) of the 37 persons transported by emergency medical services and four employees who arrived at the ED by other means. Two persons who initially were treated and discharged returned to the ED with continuing symptoms. CO poisoning is defined as carboxyhemoglobin >5% for nonsmokers and >10% for smokers or those whose smoking status is unknown (*2*). Median age of those for whom medical records were obtained was 27 years (range = 20–63 years), 16 (40%) were female, and 15 (38%) smoked or had undocumented smoking status. The most commonly reported symptoms were headache, dizziness, and nausea, which were reported by 37 (93%), 16 (40%), and 15 (38%) patients, respectively. Mean blood carboxyhemoglobin level among 37 (93%) workers evaluated within 6 hours of the first responders’ arrival was 11.7% (range = 4.1%–21.4%). Thirty-one (78%) patients met the Council of State and Territorial Epidemiologists’ CO poisoning case definition (*2*). No patients required overnight inpatient admission or hyperbaric oxygen. There were no deaths.

An Occupational Safety and Health Administration (OSHA) investigation identified a CO source associated with gas burners on the fryer appliances; these burners had been replaced 4 days earlier. OSHA found that ventilation was inadequate to clear combustion products from the new burners, which resulted in a buildup of CO in the facility.

Gas-burning appliances in industrial kitchen facilities are not common occupational causes of CO-related morbidity and mortality (*3*). However, improperly maintained and ventilated appliances can be a source of CO exposure. Because symptoms of CO poisoning are nonspecific, CO poisoning might be underreported (*4*). Adequate ventilation in areas at risk for CO buildup, routine maintenance of gas-burning equipment, and detectors that alert to potentially unsafe levels are the best ways to prevent CO poisoning. OSHA does not specifically require CO detectors in industrial kitchen facilities; however, employers are required to evaluate all potential airborne contaminants that present a health hazard (*5*).
